# Strategies for capacity building for health research in Bangladesh: Role of core funding and a common monitoring and evaluation framework

**DOI:** 10.1186/1478-4505-9-31

**Published:** 2011-07-28

**Authors:** Shakeel Mahmood, Krishna Hort, Shakil Ahmed, Mohammed Salam, Alejandro Cravioto

**Affiliations:** 1 International Centre for Diarrhoeal Disease Research, Bangladesh (ICDDR,B), 68, Shaheed Tajuddin Ahmed Sarani, Mohakhali, Dhaka 1212, Bangladesh; 2 Nossal Institute for Global Health, The University of Melbourne, Level 4, 161 Barry Street, Carlton, Victoria 3010, Australia

**Keywords:** Research capacity building, health research, low and middle income countries (LMIC), monitoring and evaluation, and core funding

## Abstract

**Background:**

There is increasing interest in building the capacity of researchers in low and middle income countries (LMIC) to address their national priority health and health policy problems. However, the number and variety of partnerships and funding arrangements can create management problems for LMIC research institutes. This paper aims to identify problems faced by a health research institute in Bangladesh, describe two strategies developed to address these problems, and identify the results after three years of implementation.

**Methods:**

This paper uses a mixture of quantitative and qualitative data collected during independent annual reviews of the International Centre for Diarrhoeal Disease Research, Bangladesh (ICDDR,B) between 2006 and 2010. Quantitative data includes the number of research activities according to strategic priority areas, revenues collected and expenditure. Qualitative data includes interviews of researchers and management of ICDDR,B, and of research users and key donors. Data in a Monitoring and Evaluation Framework (MEF) were assessed against agreed indicators.

**Results:**

The key problems faced by ICDDR,B in 2006 were insufficient core funds to build research capacity and supporting infrastructure, and an inability to direct research funds towards the identified research priorities in its strategic plan. Two strategies were developed to address these problems: a group of donors agreed to provide unearmarked pooled core funding, and accept a single common report based on an agreed MEF. On review after three years, there had been significant increases in total revenue, and the ability to allocate greater amounts of money on capacity building and infrastructure. The MEF demonstrated progress against strategic objectives, and better alignment of research against strategic priorities. There had also been changes in the sense of ownership and collaboration between ICDDR,B's management and its core donors.

**Conclusions:**

The changes made to funding relationships supported and monitored by an effective MEF enabled the organisation to better align funding with research priorities and to invest in capacity building. This paper identified key issues for capacity building for health research in low and middle income countries. The findings have relevance to other research institutes in similar contexts to advocate and support research capacity strengthening efforts.

## Background

Strengthening research capacity is considered one of the most powerful, cost-effective, and sustainable means of advancing health and development [1]. In the last two decades, there has been an increased interest in building and assessing health research capacity in developing countries [2-10]. Capacity for health research is defined as 'an ability of individuals, organisations, or systems to perform and utilise health research effectively, efficiently, and sustainably' [11]. This definition includes research capacity at the level of individuals (training), organisations and systems.

According to Nchinda [2002], research capacity strengthening consists of two main, closely inter-related and inter-dependent activities: providing institutional support, and improving researcher capacity through appropriate training. Building research capacity requires political will and credibility, a responsive capacity-building plan, leveraging, and cost sharing [2,12,13]. Research capacity is still one of the constraints that prevents institutions in developing countries from engaging in effective and essential research [5]. Building capacity for health research is a complex and long-term process [1]. Inadequate support, poor management and negative influences associated with other external or internal factors can damage the research capacities of institutions [1]. One of the factors required for effective research infrastructure is the provision of appropriate facilities that support researchers [14]. Financial and intellectual supports are also essential for building effective and sustainable research institutions [15].

Trostle and Simon (1992) argued that research capacity building is a development goal influenced by structural constraints and cultural impediments among both donors and recipients. Strengthening research capacity is mainly an initiative of donor agencies located in developed countries [17,18]. However, the WHO, the Commission on Health Research for Development, the Global Forum on Health Research, the International Clinical Epidemiology Network and other international agencies continue to emphasise the need to strengthen capacity of research institutions in developing countries [12,19-24].

A number of authors have highlighted the importance of a favourable and conducive enabling environment for research, in addition to good physical infrastructure [1,10,25-27]. This includes factors such as: adequate funds for researcher and staff salaries; training of individual researchers; career structure for researchers; good research management; equitable access to scientific and technical information; partnerships between developed and developing countries; establishment of effective interfaces between research producers and users; and competent and motivated institutional leaders. These are the key challenges to building research capacity in developing countries.

Strengthening research capacity in developing countries is heavily dependent upon external investment [24]. Lansang and Dennis (2004) identify the need for greater investment in research capacity building in developing countries, and irrespective of the source, 'a good proportion is allocated to capacity building'. They suggest the need to 'document and analyse success and lessons learnt from countries and organisations that have devised innovative financing schemes for health research'.

Mayhew *et al*. (2008) concluded that capacity building cannot be achieved without substantial financial input and recommended that development partners should provide long-term and in-country support for successful research capacity building. Development partners need to be focused and make coordinated efforts to achieve maximal effect. Further research is needed to identify the best funding support to build capacity for research [28].

Simon J (2000) supported strengthening health research capacity building in developing countries. He pointed out that that there is little information available on the impact of millions of dollars of investments for building research capacity and indeed, little has been written on how research capacity development efforts can be monitored and evaluated. There is a lack of agreed indicators for measuring progress or achievement, which are crucial to evaluate the impact of investments on research capacity. There is also a need to document the results and impacts of different kinds of investment for development partners and policy makers [29].

### ICDDR,B

The International Centre for Diarrhoeal Disease Research, Bangladesh (ICDDR,B) is a well established international health research institute focusing primarily on research that offers data for a wide range of disease areas to help decision makers, especially in public health. Although the information published and disseminated by ICDDR,B has global utility, it specifically focuses on health issues that are more relevant to low income countries. Since its establishment in 1960, ICDDR,B has been the recipient of significant amounts of international donor funding to support its development and research projects. Over the past 50 years, the organisation has made significant contributions to expanding knowledge and service delivery practice in health; in particular the development and introduction of oral rehydration solution (ORS) for severe diarrhoea.

Despite valuable and constant financial support, a review in 2006 observed that ICDDR,B faced a number of challenges in managing international funding in terms of maintaining sustainable development and sustained research contributions. The following article aims to assess whether the interventions in management of donor support implemented in response to the review had enabled ICDDR,B to satisfactorily address these problems after three years

## Methods

This research is based on a mixture of qualitative and quantitative methods, using a case study approach. Qualitative data include key informant interviews as well as a series of questionnaires with managers and key researchers at ICDDR,B; with other research collaborators; key funders; and potential users of research output, including policy makers within the Ministry of Health, Government of Bangladesh, and other national and international development organisations operating in the health sector. The research output from the organisation was assessed in terms of coverage of identified research priority areas, and the extent to which the research addressed different phases of the development of interventions: basic and epidemiological research; causative research; intervention research; and evaluation of implementation of interventions.

Quantitative data included counts of research outputs and publications, patients treated at ICDDR,B's clinical facilities, and numbers attending training courses and sessions. It also included an analysis of ICDDR,B's finances, including income and expenditure. Data sources included: (a) Annual reports published by ICDDR,B for the years 2006-2010; (b) Monitoring and evaluation reports prepared by the Monitoring and Evaluation Unit of the organisation based on the indicators in the agreed Monitoring and Evaluation Framework (years 2007-2009); and (c) Annual external reviews of ICDDR,B conducted by independent consultants for the years 2006 to 2009. (Author KH was the independent consultant for the reviews of 2006 and 2010). During the reviews, qualitative data was collected through interviews, and the assessments and reports prepared by the ME Unit of performance against MEF indicators were reviewed. A summary of study areas of focus and data sources is provided in Table 1

**Table 1:** Areas of study, methods and data sources

Areas of Study	Methods	Data Sources
Context: perceived problems and issues	Qualitative	Interviews with ICDDR,B management; key donors; principal researchers

	Quantitative	Measurement of research output and topics; Financial analysis

Intervention	Qualitative	Interviews with ICDDR,B management; key donors; principal researchers Discussion with M&E unit

	Quantitative	Assessment of data provided against M&E indicators

Impact of intervention; changes within ICDDR,B	Qualitative	Interviews with ICDDR,B management; key donors; principal researchers Discussion with M&E unit; Discussion among authors

	Quantitative	Measurement of research output Financial analysis

Impact externally	Qualitative	Interviews with Government of Bangladesh policy makers; collaborating research institutes; other national and international development agencies in Bangladesh

The ICDDR,B Monitoring and Evaluation Unit was established in August 2007 with one coordinator and a core team of four assistants liaising with designated divisional staff who facilitate the compilation of requisite information from the Principal Investigators (PIs). The Unit collects, compiles and reports the data required to assess the performance indicators included in the MEF. Data were collected via a series of questionnaires developed by the MEF unit and entered by the designated divisional staff onto a computer entry form.

Collated data were then meticulously analysed, modified or deleted by the MEF coordinator with the agreement of the respective PI to avoid confounders and the resulting information applied to the MEF tables. A final scrutiny of the tables was carried out by the directors of one of ICDDR,B's four scientific divisions before sharing with the core donors. From the pilot MEF in 2006, this process was repeated each year and reports registered and archived. The 2010 review evaluated the reports from 2007, 2008 and 2009.

## Results

### Scope of activities undertaken by ICDDR,B

ICDDR,B is an international health research organisation located in Dhaka, Bangladesh. Founded in 1960 as the Pak-SEATO Cholera Research Laboratory, it was established as an international organisation by an ordinance of the Government of Bangladesh in 1978. It was originally focused on cholera and other diarrhoeal diseases, and related problems of nutrition and family planning. Since then, its scope has broadened to include many of the major public health disciplines and issues facing poor countries: child health, infectious disease and vaccine sciences, reproductive health, nutrition, population sciences, health systems research, safe water, food safety, HIV/AIDS and poverty and health [30].

ICDDR,B is primarily a research institution, and it maintains ongoing surveillance in regularly monitored urban and rural communities, as well as conducting laboratory research. It has a particular focus on a research pathway of initial epidemiological research to identify changes in population health; in depth studies to identify causes and develop interventions; operational research on the implementation of interventions and evaluation studies of their effectiveness. Examples include the development of oral rehydration solution, and zinc treatment for diarrhoea.

ICDDR,B also provides training in topics such as research methods, clinical management of diarrhoeal disease, laboratory methods, epidemiology and biostatistics, family planning and demographic surveillance both to its staff and to trainees from national, regional and international institutions and individuals.

As part of its surveillance programme, and as a contribution to the health of populations under surveillance, ICDDR,B provides clinical services in the areas of its expertise such as treatment of acute and persistent diarrhoea, nutrition rehabilitation of malnourished children, treatment of acute respiratory infections, and treatment of HIV-AIDS. The organisation also assists the agencies of the Government of Bangladesh in investigating and responding to outbreaks and epidemics of potentially infectious disease.

Governed by a distinguished Board of Trustees (BOT) comprising 17 multinational members, ICDDR,B's development is guided by a series of strategic plans, which identify research priorities, and capacity building objectives. The majority of its funding is provided as research grants for specific projects, provided by donors or through competitive process. A few donors, including the Government of Bangladesh, the Australian Agency for International Development (AusAID); the Canadian International Development Agency (CIDA); the Department for International Development (DFID) United Kingdom; the Embassy of the Kingdom of the Netherlands (EKN); and the Swedish Agency for International Development Cooperation (SIDA), provide funding for specific infrastructure and capacity building activities, not directly linked to research (core donors).

### The 2006 Annual Review and Proposed changes

During the 2006 annual review of ICDDR,B, a number of issues were identified, which were considered important for the future development of research-based funding.

ICDDR,B was receiving funding from around 55 different donors and research granting bodies, each with their own specific research interests, financing rules and regulations, and reporting requirements. At the same time, the organisation's need for funding the underlying research infrastructure and administrative and management systems (denoted as "core activities") was growing. In order to finance infrastructure development required to maintain research projects for donor organisations, ICDDR,B requested a 30% management levy from the research donors. However, many were reluctant to make this contribution and, in addition, this levy proved unpopular with the organisation's researchers who felt that it made their proposals less competitive. Only a few donors were prepared to contribute 'unearmarked' funds or funding for specific aspects of core activities.

ICDDR,B management felt that it was constrained in pursuing the research priorities identified in its strategic plan and was being tied to the interests of research donors. Furthermore, the organisation's capacity to invest in developing and maintaining the physical and human resource infrastructure needed to support research was limited.

In consultation with the organisation's management team and the small group of donors prepared to invest in core activities, referred to as the Core Donor Group (CDG), the 2006 review proposed a change in the way ICDDR,B and its CDG interacted. This change involved the following: the core donors agreed to provide 'unearmarked' funding to ICDDR,B to support its infrastructure development and maintenance, and capacity building; the organisation was required to report to the core donors on the actual use of these funds through a single annual financial report, and a single comprehensive report of progress in achieving the agreed indicators using a common Monitoring and Evaluation Framework (MEF); and jointly, the organisation and its core donors were to undertake an annual review of core fund use reporting against the indicators in the MEF.

### Monitoring and Evaluation Framework (MEF)

The development of the MEF was a key requirement for the funding changes to be made and it provided the agreed framework for core donor commitment, as well as directing the future development of ICDDR,B, and creating a tool allowing for measuring the progress. The main challenge was in reconciling the interests and priorities of the donors with those of ICDDR,B. The organisation's strategic plan (2001-2010) at that time focused on identifying priority research areas, and provided little guidance on the directions or extent of capacity or infrastructure development, or on the expected outcomes from research in those identified priority areas. The CDG members were particularly interested in ensuring that research addressed key policy issues for health systems in low and middle income countries (LMIC), and that the research findings were communicated to policy users and, where appropriate, influenced policy decisions.

The MEF, which was developed and agreed in 2007, addresses the four key areas of ICDDR,B's commitment: research, clinical services, teaching, and improvements in management and operations. For each area, priority outcomes, outputs and activities are nominated, with indicators providing suitable measures. Where possible, indicators are based on data already being collected at ICDDR,B, or where the organisation has the capability to collect the required data. Where available, the outcomes and outputs in the MEF refer to the priorities and development targets outlined in the Strategic Plan (Table 2), The MEF approach was cited with approval in Morris et al 2008 and this can become a key element of performance reporting for organizations [31].

**Table 2:** Summary of outcomes and outputs from MEF

Level	Research	Clinical Services	Training	Operation & Management
Outcomes	New knowledge relevant to global and local health needs Changes in programs, policies and practices which incorporate this knowledge	Contribution to health of populations served Knowledge of service needs & clinical interventions	Local and regional health/research workforce have improved skills and knowledge in relevant areas Incorporation of knowledge into practice	Growth and development of ICDDRB in line with vision and mission Maintain relevance of ICDDRB to local, regional and national context

Outputs	Research agenda which identifies priority research areas & objectives Program of research activities which is: (a) in line with priorities in strategic plan (SP) (b) sensitive to issues of gender, environment, poverty, & equity (c) credible and high quality based on sound methodology & innovation (d) conducted according to ethical standards with benefit for research participants Users and decision makers in relevant areas informed of research findings & implications	Effective treatment is provided to patients Equity in access to services appropriate to need Efficient use of resources in provision of services Quality of clinical services maintained and risks to patients and staff minimised Research results and findings relevant to clinical services	Training program based on workforce needs and ICDDR,B areas of contribution Course participants have knowledge and improved skills in relevant areas Quality: Knowledge and skills based on relevant evidence and effective teaching methods Increase in total and externally provided funds for training.	Governance and decision making provides effective leadership Efficient & effective financial management Effective management of partnerships with donors Effective management and development of human resources Maintenance and development of facilities and infrastructure Effective information management

### The 2010 Annual review and changes identified

The annual external review of 2010 was extended to assess the effect of the changes recommended in 2006 on ICDDR,B's performance over the period 2007 to 2009 against the MEF indicators, and to evaluate, in more depth, the contribution made by core funding to the research carried out by ICDDR,B. The review identified the following changes:

### Changes in the relationship between core donors and the ICDDR,B

Both the core donors (CD) and ICDDR,B management reported a significant 'qualitative' change in the relationship between each other. The CD functioned more as a coherent group, rather than individually, facilitating collective communication and decision making. Frequent communication and a good relationship with the chair of the group of CD dramatically reduced the need for multiple communications to each individual donor.

There had been a genuine attempt to maintain the commitment to a single combined funding, and monitoring and evaluation (M&E) process, despite some reservations. The single combined financial reporting had been largely accepted, as well as the joint annual M&E review.

ICDDR,B management reported that the reduction in reporting from quarterly to annual, and a single rather than multiple-reporting format for the core donors had reduced transaction costs, and led to further progress towards harmonised and convergent management and funding processes. From this collaborative approach on behalf of the CD, a single consolidated funding request process has been established. Core donors also reported that the annual reporting process and single pooled funding contribution reduced their own costs in oversight and supervision.

Interviews with ICDDR,B managers noted a marked change in terms of ownership and control, with ICDDR,B demonstrating much greater capacity and ability to control its own development, and preparedness to invest core funds particularly in neglected areas in order to build future capacity. This had not been possible in the past, when development of research programmes and capacity had been mainly 'donor driven'.

The success of the first three years of the CD and MEF has led to the development of a new strategic plan for the period 2011-2020, which capitalizes on the positive outcomes of this new funding approach and incorporates the organisation's priorities into the new strategic plan with improved monitoring, evaluation and reporting structures and processes.

### Use of the MEF

The MEF has been used as the basis for annual reporting and reviews for the years 2007, 2008 and 2009, with modifications being made on an on-going basis in line with developments in the collection and compilation of data, and new developments in services and research interests.

The quality of reporting and the information provided in the MEF report has improved progressively, and in the 2010 review, the 2009 data was either good or sufficient in terms of performance assessment against 80% of related indicators. The main weaknesses in reporting were excessive detail, lack of focus on priority areas or significant results, and lack of comparative analysis. Reporting against outcomes, particularly in the areas of research and clinical services, was found to be poor, and recommendations were given as a means to strengthen this element.

However, the interviews found some differences in the perception of managers and researchers (especially principal investigators) regarding the MEF. While managers saw the benefits in terms of savings in transaction costs, and greater autonomy in use of funds, researchers tended to view it as an additional donor requirement and of little benefit to them personally. It was clear that some of this alienation was due to ICDDR,B's existing structure. With this in mind, the new Strategic Plan 2020 includes a significant restructuring to better align researchers with the strategic priorities of the organisation, and their reporting with the MEF.

### Performance in key areas of research, clinical services, training and capacity building, management, and operations

The review found that ICDDR,B has continued to perform well in all areas (Table 3). In terms of research, the review documented significant research findings, and incorporation of these findings into international policy and national programme guidelines in five of the priority research areas, as described in the Strategic Plan, 2001-2010. In particular, the interviews with external stakeholders identified that ICDDR,B researchers employed a range of mechanisms for disseminating information and policy engagement, and that building relationships between researchers and policy makers had been key to influencing policy in the complex and dynamic political environment of Bangladesh.

**Table 3:** Research outcomes and key indicators and achievement

Outcome/output	Indicators	Achievement (2009)
Outcome		

1. New knowledge relevant to global and local health needs	Implications of research findings or results for current knowledge/understanding or programs/interventions in relation to MDGs or national priorities.	Review of progress and policy development demonstrates significant contributions in 6/8 priority areas of Strategic Plan 2001-2010

2. Changes in programs, policies and practices which incorporate this knowledge	Changes in national/regional or international policy/programs consistent with new knowledge/research findings.	Significant policy changes/policy contribution in 5/8 priority areas of Strategic Plan 2001-2010

Outputs		

1. Updated research agenda which identifies priority research areas & objectives	Revisions to the priority research areas based on consultation with stakeholders and research findings undertaken at least every 2 years.	Strategic Plan 2010-2020 provides information on new research directions and justification

2. Program of research protocols and research activities which contribute to the objectives in the priority research areas identified in the Strategic Plan	Number of new research protocols/activities approved by research priority area and research phase during the last 12 months	Increase in infections (27%), health systems (17%); decrease in diarrhoea (9%) and HIV (8%) Shift towards operational research

3. Research program addresses issues of gender, environment, poverty and equity where relevant.	No/% of protocols approved during the last 12 months which address - gender, environment, poverty and equity.	Very low proportions - no significant change over 2007-2009 periods. Reporting is not capturing this information.

4. Research undertaken by ICDDR,B is regarded as high quality and innovative by international peers.	No. of publications in the high impact peer reviewed journals by research priority area during last 12 months.	Most in traditional strong areas of diarrhoea (1/3), infections, child health

	No. of citations in peer reviewed literature for key publications in each priority research area over a 5 year period.	Citation rate by area: highest in diarrhoea (7.3); infectious disease (4.9); child health (4.5)

5. Research undertaken by ICDDR,B is conducted according to ethical standards	Presence of policies on scientific misconduct, acknowledgement, authorship, sex trafficking, allocation core funds	Policies established, but compliance not known

	Numbers and proportion of investigators certified for human research	Proportion has risen to 97% from low levels in previous years

The number of patients treated through clinical services increased while there were also improvements in the range of services and standard of care provided. Increases in the number and range of training provided, particularly for ICDDR,B staff and for GoB agencies, were also highlighted in the review. The number trained in research skills increased from 36 in 2007 to 120 in 2009 with 40% of participants being female, while the number trained in clinical skills remained between 120 and 130 per year, with the proportion of female trainees increasing from 20% to 30%.

In the area of management and operations, ICDDR,B achieved two of the six priorities identified in the 2001-2010 Strategic Plan, namely reaching the financial target of US$30 million annual gross income; and the introduction of a computerised financial management system. The remaining four priorities appeared to be making significant progress, including introduction of IT applications, extension of the main ICDDR,B building to 8 floors, and transition in HR management from the UN system to a competency-based system.

There was significant progress in the area of human resource management, with the establishment of an HR development unit, the development and implementation of a specific gender policy, an assessment of organisation-wide training needs, and the expansion of training provided to staff in identified priority areas: English language; computer applications; and scientific writing.

### Contribution of core funds to ICDDR,B

Against continuing increases in total funding for the organisation, which increased from $23.15 million to $38.69 million between 2006 and 2009, the proportion of total revenue represented by core funds increased from 25% to nearly 40%, a figure that has since remained relatively steady. Total core donor funding in 2009 was $14,099,000 of which the CD contributed $13,070,000 (94%) At the same time research grant and project income has also increased, from $13.3 million in 2006, to $23.1 million in 2010, an increase of nearly 75% (Table 4).

**Table 4:** Revenue and expenditure by year (USD '000)

Revenue type	2006	2007	2008	2009
Research projects	13,307	16,674	20,906	23,065

Un-earmarked core funds	7,205	10,393	11,782	14,099

Other*	2,638	1,820	1,953	1,530

Total**	23,150	28,887	34,642	38,694

Operating Expenditure**				

Research projects	13307	16674	20906	23065

Core funded research	301	895	804	964

Clinical services	2238	2747	4315	5323

Management systems	2747	4019	2906	3292

Infrastructure/research support/capacity building	3390	5227	5179	5718

Total	21983	28756	34450	38598

*Includes management levy from research funding

** Excludes capital expenditure.

There were some changes in relationships with other donors that contributed to this improved financial performance, including agreement by the Government of Bangladesh to provide funding for new capital works, and to channel funding from the Health Sector Support Project to ICDDR,B; and relaxation in the requirements for funding from USAID that enabled ICDDR,B to receive USAID funding after a period when family planning restrictions had prevented it.

Total core expenditure, including core capital expenditure, increased by 31% between 2007 and 2009. The amount contributed by core donor funding rose slightly from 78% in 2007 to 81% in 2009; the remaining 19-20% was derived from management levies, research project funds, and other receipts. The 80% core expenditure covered by CD funds comprises a combination of operational/recurrent expenditure, plus investment in new systems, processes and equipment (Table 4).

Core funds have also been used to support research in new areas while research proposals and capacity building are being encouraged and developed (e.g. in health systems). Direct funding of research from core funds remains relatively small at only 5% of total research funds, and focuses more on those areas for which research grants are difficult to attract; strategic pilot or 'seed' research, which has attracted grants at a later stage; and building capacity of young career researchers.

Principally, core funds were allocated to build research infrastructure, in terms of systems, human resources, and equipment. This is a key area in which it was previously difficult for ICDDR,B to invest, due to the constraints imposed by specific research project funding. However, the core contribution has significantly improved ICDDR,B's future capacity to retain and develop new researchers, and to compete for and gain competitive research grants

## Discussion

While this review only documented changes over a three year period, it suggests that the changes to the funding and reporting arrangements between the core donors and ICDDR,B has had a significant impact on the problems identified in the 2006 review.

In particular, ICDDR,B management feels that the new funding arrangement has increased autonomy to pursue its identified strategic priorities; has enabled the organisation to invest in research infrastructure and workforce capacity development; and has maintained its success in obtaining research grants and project funding. These investments have focused on human resource development at ICDDR,B, which is consistent with the literature, with development of 'the capabilities of scientists to undertake quality research' being one of the two elements of research capacity building identified by Nchinda [10].

The use of core funds as 'seed' funds for developing research proposals is one way that ICDDR,B has addressed the problem alluded to by Sitthi-amorn and Somrongthong [9] as "the 'brain drain' of scientists and researchers to developed countries". The seed funds enabled returning PhD students to commence their careers as researchers, without having to wait until they could win research grants through their own initiatives.

There have also been efficiency savings in terms of reduction in multiple reporting and communication with multiple donors, and continued growth in total revenue from both research grants and core funding. While other changes in the operating context such as the increased funding from the Government of Bangladesh and USAID clearly contributed, it is likely that the increased investment in infrastructure and human resources assisted ICDDR,B in responding to these new opportunities.

The effectiveness of the core funding mechanism is dependent on a common and agreed strategy, and an effective MEF to measure, monitor and evaluate progress in achieving the strategic elements. Establishing such a framework in the context of a research institute is challenging, as researchers traditionally value autonomy in pursuing their research objectives, rather than being guided by institutional strategy and key performance indicators.

While the principal focus of the MEF has been on reporting to donors, ICDDR,B management have been keen to use the MEF to improve monitoring and reporting within the organisation on achievement of the Strategic Plan priorities. The establishment of the M&E Unit, and the regular annual reviews have imposed additional costs on both ICDDR,B and donors, but also enabled them to periodically revise and improve the MEF.

Authors of other published articles have commented on the difficulty of identifying appropriate indicators for measuring research capacity building. Commonly measured outcomes include publications in peer-reviewed journals, successful grant applications, qualifications of the researchers, projects per year, projects per researcher, project duration of greater than one year, and conference presentations [3,25,28,32]. As Cooke (2005) noted, these 'traditional outcomes may not address all the relevant issues to highlight progress' in research capacity building. In particular, she notes the need to address the supportive environment, usefulness or social impact of research, and professional outcomes [32].

The experience at ICDDR,B confirms the importance of an agreed MEF, with indicators that capture the full breadth of the organisation's activities, and are sensitive to the capacity building efforts. The elements of the MEF reflect the six principles proposed by Cooke (2005) [32]: developing skills and confidence; supporting links and partnerships; ensuring that research is 'close to practice'; developing appropriate dissemination; investing in infrastructure; and building elements for sustainability and continuity. ICDDR,B's clinical services provide the opportunity for research that is 'close to practice', while the management and operations' section addresses infrastructure, and sustainability and continuity.

## Conclusions

While this paper reports on the experience of one research institute in a developing country context, there are three key issues which we believe are of relevance to other institutes in similar contexts:

(1) Investment in capacity building and maintaining physical and human resource infrastructures of research institutes is vital to their sustained operation. However, adequate funding for these needs cannot be obtained through funding for specific research projects or through research grants, even with a 'management levy'.

(2) Part of this capacity is the ability and autonomy of the research institute to determine its own strategic growth and research priorities, and to invest in pursuing these, rather than the priorities determined by research funders.

(3) The provision of unearmarked core funding for use by the research institute to invest in the agreed strategic priorities is a potential mechanism to build this capacity and to maintain this autonomy. In many ways, this situation is comparable to that of the governments of low income countries when dealing with donors, with the process established at ICDDR,B has been likened to a 'mini SWAPs (Sector Wide Approaches)', consistent with the principles of the Paris Declaration on Aid Effectiveness [33].

However, as in SWAps, there is a need for significant capacity on the part of the research institute to manage and operate such a mechanism. This includes the development of its own strategic plan and identification of investment priorities; the ability to manage and report on use of the funds in a financially accountable manner; and the ability to report on results through an agreed M&E framework. Underlying this is the need for confidence and a strong relationship between the research institute and its donors, that allows for robust dialogue and the resolution of differences.

ICDDR,B has been fortunate in having a long period of interaction with donors prior to the establishment of this mechanism in which to build up this capacity, and to develop these relationships.

## Competing interests

SM, MS & AC work at the ICDDR,B. They contributed to the development of the MEF and the collection and reporting of MEF data over the years 2007 to 2009. They have in-depth knowledge of ICDDR,B and MEF and their contribution added value to the evaluations. There is no opportunity for these three authors for competing interests. KH was funded for this evaluation study by SIDA Bangladesh Office. However SIDA had no influence on the analysis of the evaluation. This paper does not represent SIDA or CD views. SA declares that he has no competing interests.

## Authors' contributions

KH designed and conducted the 2010 evaluation. KH contributed to the manuscript structure, and to the methods, results and conclusions sections. SA conducted literature review and contributed to the background section and overall editing and preparation of the manuscript. SM, MS and AC contributed to the manuscript structure and drafted the sections: ICDDR,B - International Centre for Diarrhoeal Disease Research, Bangladesh and its role in capacity building for health research; the 2006 annual review and proposed changes; monitoring and evaluation framework and use of the MEF. They also contributed to review and editing of text. All authors read and approved the final manuscript.

## Authors' information

SM, MS & AC: International Centre for Diarrhoeal Diseases Research, Bangladesh (ICDDR,B), 68 Shaheed Tajuddin Ahmed Sarani, Mohakhali, Dhaka 1212, Bangladesh.

KH & SA: Nossal Institute for Global Health, the University of Melbourne. 161 Barry Street, Carlton, Vic 3010, Australia.
